# Spikelets in Pyramidal Neurons: Action Potentials Initiated in the Axon Initial Segment That Do Not Activate the Soma

**DOI:** 10.1371/journal.pcbi.1005237

**Published:** 2017-01-09

**Authors:** Martina Michalikova, Michiel W. H. Remme, Richard Kempter

**Affiliations:** 1 Institute for Theoretical Biology, Department of Biology, Humboldt-Universität zu Berlin, Berlin, Germany; 2 Bernstein Center for Computational Neuroscience Berlin, Berlin, Germany; University of Hertfordshire, UNITED KINGDOM

## Abstract

Spikelets are small spike-like depolarizations that can be measured in somatic intracellular recordings. Their origin in pyramidal neurons remains controversial. To explain spikelet generation, we propose a novel single-cell mechanism: somato-dendritic input generates action potentials at the axon initial segment that may fail to activate the soma and manifest as somatic spikelets. Using mathematical analysis and numerical simulations of compartmental neuron models, we identified four key factors controlling spikelet generation: (1) difference in firing threshold, (2) impedance mismatch, and (3) electrotonic separation between the soma and the axon initial segment, as well as (4) input amplitude. Because spikelets involve forward propagation of action potentials along the axon while they avoid full depolarization of the somato-dendritic compartments, we conjecture that this mode of operation saves energy and regulates dendritic plasticity while still allowing for a read-out of results of neuronal computations.

## Author Summary

Action potentials (APs) are digital, all-or-none signals by which neurons communicate with each other. Therefore, APs are the basis of neural function, yet some of their fundamental features are still not well understood. Here we focus on pyramidal cells, which are the principal neurons in neocortex and hippocampus. According to textbook knowledge, an AP in pyramidal neurons is initiated at the axon initial segment and propagates along the axon to the next cell. Concurrently, the AP also propagates back to the soma and into the dendrites where it might trigger synaptic plasticity, which is the basis of learning and memory. However, besides APs, pyramidal cells sometimes also show somatic spikelets—small depolarizations with an AP-like shape—whose origin remains unclear. Here, we propose that spikelets occur when an AP initiated at the axon initial segment only propagates down the axon, but fails to activate sodium currents in the soma and dendrites. As a result, spikelet firing saves energy, and moreover, might be a means to control synaptic plasticity and thereby control learning and memory.

## Introduction

Brain functions rely on computations in single neurons, but some basic features of neural processing still remain unclear. Here, we focus on spikelets, which are brief, spike-like depolarizations of small amplitude (< 20 mV). Spikelets can be measured in somatic intracellular recordings in diverse neuron types, including cortical interneurons (e.g., [1]) and pyramidal cells [2–4]. Due to their all-or-none appearance and spike-like shape, spikelets are considered to reflect action potentials (APs) occurring in electrotonically distinct compartments. These APs might originate either in the dendrites or in the axon of the same cell, or in another neuron that is either coupled ephaptically or through gap junctions. Since spikelets influence somatic voltage dynamics, including AP generation [2], identifying the origin of spikelets is important for understanding neural computations.

The origin of spikelets in hippocampal [2, 3, 5] and neocortical [4] pyramidal neurons is not well understood. The original hypothesis of spikelets resulting from dendritic spikes [6] could not be supported by subsequent studies [7]. Instead, axo-axonal [8, 9] and somato-dendritic [10, 11] gap-junction coupling of pyramidal neurons has been suggested as the spikelet origin, however, the supporting experimental evidence is scarce, raising the question whether there are other mechanisms for generating spikelets in pyramidal neurons.

*In vitro*, somatic spikelets can be evoked with distal axonal stimulation if an antidromically propagating AP [12] does not suffice to activate the somatic sodium channels. This can happen because of somatic hyperpolarization, (prolonged) somatic depolarization, or fast repeated axonal stimulation [13–16]. However, *in-vivo* inputs are usually considered to arrive at the soma orthodromically. Indeed, spontaneous antidromic spikelets (also called “ectopic”) have been identified mainly under pathological conditions, such as epilepsy [17]. Additionally, antidromic spikelets are expected to occur when neurons would be coupled through axo-axonal gap junctions [8].

Here, we present a novel hypothesis for the origin of spikelets in pyramidal neurons. Using a computational approach, we demonstrate that spikelets can be evoked orthodromically with somato-dendritic inputs, which initiate APs at the distal axon initial segment (AIS). Under certain conditions, these APs in the AIS fail to fully activate the soma and appear there as spikelets. Consequently, the possibility of a forward propagating AP without it propagating back to the soma and into the dendrites presents a powerful mechanism for control of dendritic plasticity while ensuring the read-out of neural computations.

## Results

### In vivo-like input generates spikelets in a detailed model of a cortical pyramidal neuron

**Fig 1:**
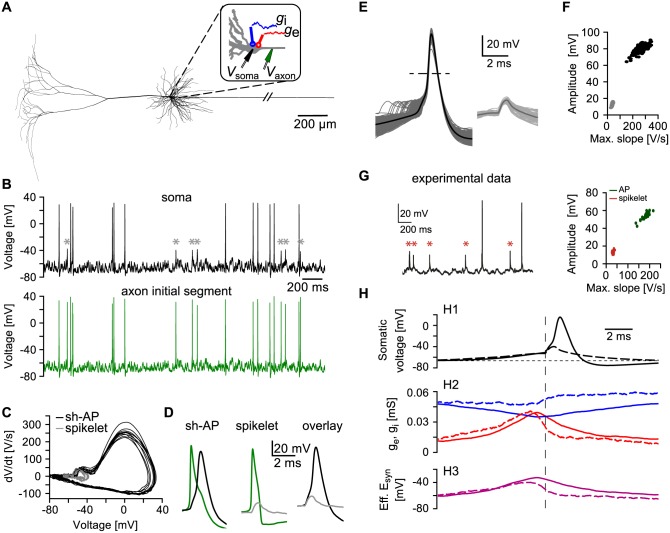
Somatic spikelets in a detailed biophysical model of a cortical pyramidal neuron in response to noisy input. A: Morphology of the model neuron. Inset: excitatory (*g*_e_, red) and inhibitory (*g*_i_, blue) conductances are placed at the soma. Recording electrodes are placed at the soma (*V*_soma_, black) and the AIS (*V*_axon_,green). Basal dendrites were removed for clarity. B: Example three seconds of membrane voltage recorded at the soma (upper trace, black) and AIS (lower trace, green) during noisy stimulation. Somatic spikelets are marked with gray asterisks (*). Spikelets co-occur with APs at the AIS. C: Phase plot of ten somatic APs (black) and ten somatic spikelets (gray). D: Examples of a somatic AP (left, black) and a somatic spikelet (middle, gray) overlaid with the corresponding APs at the AIS (green traces). Right: overlay of the somatic AP (black) and the spikelet (gray). E: All somatic events generated during a 100 s simulation. Left: APs (*N* = 579, dark gray), aligned in time to crossing of the somatic voltage threshold (-10 mV, dashed line). The mean is shown in black. Right: spikelets (*N* = 63, light gray), aligned to the voltage threshold (-10 mV) crossing at the AIS. The mean is shown in dark gray. F: The all-or-none nature of APs (black) and spikelets (gray) is revealed in a plot of event amplitude against the maximum slope. G: Left: an example voltage trace recorded in a CA1 pyramidal neuron in a freely moving rat. Spikelets are marked with red asterisks (*). Right: Event amplitude plotted against the maximum slope of APs (dark green) and spikelets (red). Adapted from [2]. H: AP- and spikelet-triggered averages (solid and dashed lines, respectively), aligned to the time of crossing the voltage threshold in the AIS (vertical dashed line). H1: mean somatic AP (solid line) and mean somatic spikelet (dashed line) waveform. The horizontal dashed line accentuates the depolarization prior to AP and spikelet occurrence. H2: mean excitatory (red) and mean inhibitory (blue) AP-triggered (solid line) and spikelet-triggered (dashed) conductances. H3: the mean effective synaptic reversal potential combines mean excitatory and inhibitory conductances (see also Methods). During APs (solid line), the synaptic drive was stronger than during spikelets (dashed line).

To investigate mechanisms underlying spikelet occurrence, we first used a previously published multi-compartmental model of a reconstructed layer V pyramidal neuron ([16]; Fig 1A). This model includes a detailed sodium channel distribution at the AIS and a hyperpolarized voltage shift of 13 mV in the activation and inactivation functions of the low-threshold Na_V_1.6 channels, present in the AIS and axon. To increase the incidence of spikelets, we modestly reduced the density of sodium channels (see Methods for details). The model cell was stimulated at the soma with stochastic excitatory and inhibitory synaptic point conductances [18] representing *in vivo*-like background activity. The resulting somatic voltage traces (Fig 1B, top) showed both APs and spikelets (stars). All APs were shoulder-APs (sh-APs; [2]) characterized by two components in the rising phase. The first component (the shoulder) was slower and resembled the waveform of spikelets (Fig 1C); the second, faster component included the peak of the AP.

**Fig 2:**
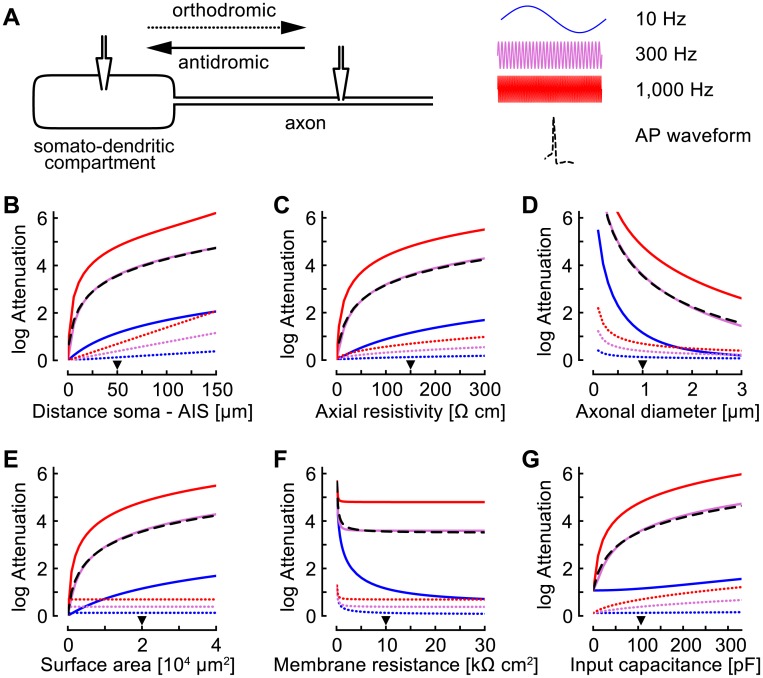
Signal attenuation in a passive-membrane model. A: The model consists of a somato-dendritic compartment attached to a semi-infinite cable (axon). Attenuation of sinusoidal inputs was calculated according to equations given in the Methods. Attenuation of an AP waveform was determined numerically. B–G: The natural logarithm of attenuation is plotted for the antidromic, axon-to-soma (solid lines) and for the orthodromic, soma-to-axon (dotted lines) signal propagation for three input frequencies: 10 Hz (blue), 300 Hz (purple), and 1,000 Hz (red). The results for the antidromic propagation of an AP waveform are shown as black dashed lines. The triangle indicates the default value of the parameter that is varied, all other parameters are held constant at their default values (see Methods for the default parameter values). The attenuation was determined in dependence upon the following model parameters: physical distance between the stimulation and the recording sites (B), axial resistivity of the axon (C), diameter of the axon (D), surface area of the somato-dendritic compartment (E), specific membrane resistance (F), and input capacitance of the somato-dendritic compartment (G), which was varied selectively by changing the specific membrane capacitance of the somato-dendritic compartment (range 0.01–3.1 *μ*F/cm^2^).

To reveal the origin of spikelets and sh-APs in our model, we compared voltage traces in the soma and the AIS (Fig 1B). The APs and spikelets recorded at the soma were initiated as full APs at the distal AIS (Fig 1D). Accordingly, both the shoulders of the sh-APs and the spikelets reflected axonal APs invading the soma [14, 19]. Next, we aligned APs to the times of crossing a voltage threshold in the soma, and spikelets to the times of crossing the same voltage threshold in the AIS (Fig 1E, see also Methods). This alignment revealed a variable delay between the shoulder and the peak of the AP (Fig 1E, left) and demonstrated the all-or-none nature of the spikelet waveform (Fig 1E and 1F), as observed experimentally (Fig 1G; [2]).

To understand why APs initiated at the AIS sometimes failed to elicit a somatic AP, we calculated both AP-triggered and spikelet-triggered averages of the synaptic input (Fig 1H). Excitation slowly increased ca. 5 ms before the onset of both APs and spikelets but dropped sharply prior to spikelet initiation; inhibition was stronger during spikelets compared to APs (Fig 1H2). Together, this input resulted in a weaker and briefer depolarizing synaptic drive for the initiation of spikelets compared to APs (Fig 1H3). We found that fast sodium channel inactivation, known to modulate spiking thresholds [20], was not a major factor influencing spikelet generation in our model (S1 Fig).

Spikelets can thus be generated in a computational model of a single pyramidal neuron experiencing *in vivo*-like synaptic input: APs initiated at the AIS may fail to activate the soma and appear there as spikelets.

### The soma-axon asymmetry shapes signal propagation in a passive-membrane model

Failure of AP propagation from the AIS to the soma (Fig 1) suggests that there is a strong voltage attenuation from axon to soma such that the somatic voltage does not reach the spiking threshold. To identify cell properties that could underlie such attenuation, we mathematically analyzed a passive-membrane model consisting of an axonal cable connected to a single somato-dendritic compartment (Fig 2A; see Methods for details). In particular, we computed the attenuation for sinusoidal input currents at several frequencies as a function of all model parameters (Fig 2B–2G; see Methods for equations).

A central factor influencing signal attenuation is the electrotonic distance between the soma and the AIS. Attenuation thus increases with increasing physical distance (Fig 2B), increasing axial resistivity (Fig 2C), and decreasing axonal diameter (Fig 2D). Importantly, the attenuation is typically much larger in the antidromic (axon-to-soma) than in the orthodromic (soma-to-axon) direction because the large somato-dendritic compartment provides a substantially stronger current sink for the passively propagated signal than the thin axon, i.e., there is a strong impedance mismatch between the two. Consistently, increasing the somato-dendritic surface area increased the attenuation of the antidromic signal whereas it did not affect the orthodromic propagation (Fig 2E). However, this did not reveal the nature of the current sink since the membrane resistance and the membrane capacitance are co-varied when changing the surface area. The specific membrane resistance, when varied separately in a range realistic for a pyramidal neuron (> 1 kΩ cm^2^), did not influence the antidromic attenuation for frequencies > 100 Hz (Fig 2F); in contrast, the antidromic attenuation of high-frequency (> 100 Hz) inputs was strongly influenced by the membrane capacitance (Fig 2G). For a fast, transient signal such as an AP, particularly the high-frequency components determine its shape. Indeed, in our model, the axon-to-soma attenuation of an AP waveform (black dashed lines in Fig 2B–2G) was very similar to the attenuation of a 300 Hz sine wave.

Hence, apart from the electrotonic distance between soma and AIS, the capacitance of the somato-dendritic compartment strongly influences the attenuation of APs propagating from axon to soma. In general, the attenuation is asymmetric, i.e., much larger in the axon-to-soma than in the soma-to-axon direction, which constitutes a favorable condition for spikelet generation.

### Spikelets, shoulder-APs, and full-blown APs in an active model with reduced morphology

**Fig 3:**
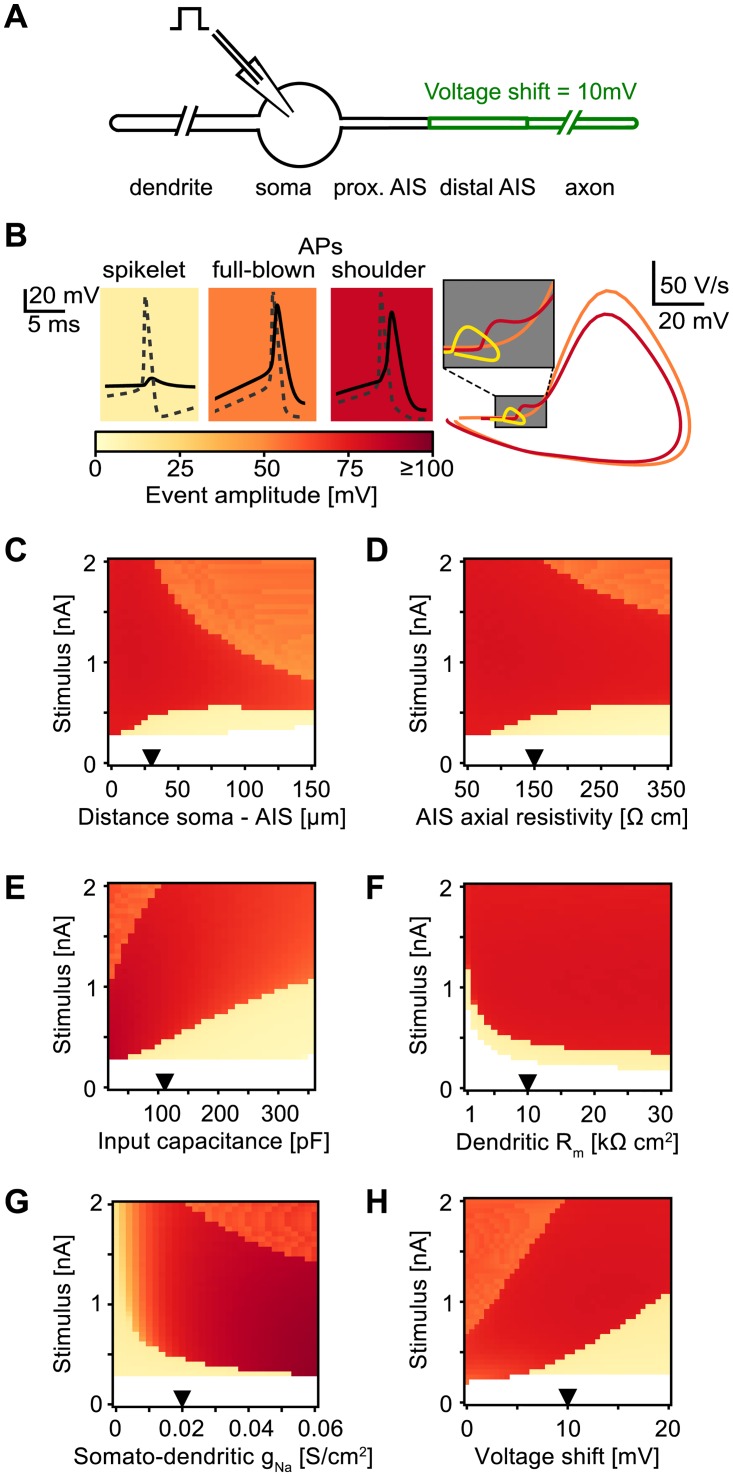
Conditions of spikelet generation in an active model with reduced morphology. A: Schematic of the neuron model. B: Left: exemplary APs and spikelets (solid line: soma, dashed line: AIS). The color bar indicates voltage amplitudes of somatic events. Right: phase plots of the exemplary somatic events shown on the left. Inset: a rapid onset (“kink”) is present for spikelets (yellow) and sh-APs (red), but not for fb-APs (orange), which arise smoothly from the baseline. Note that fb-APs reached similar maximum voltages as the sh-APs, but fb-AP amplitudes were smaller because the maximum curvature, used to define the AP onset, occurred at more depolarized voltages (see Methods for details). C–H: Amplitude of somatic events (APs or spikelets) plotted in color code as a function of the stimulus strength (ordinate) and one of the model parameters (abscissa). Default values are indicated with triangles and given in the Methods. C: Physical distance between the soma and the distal AIS. D: Axial resistivity in the proximal and distal AIS. E: Input capacitance at the soma, varied through the specific membrane capacitance (range 0.2–3.2 *μ*F/cm^2^). F: Specific membrane resistance, varied only in the dendrite. G: Sodium channel density at the soma and the dendrite. Axonal channel densities were kept constant. H: Voltage shift in the activation and inactivation curves between the somato-dendritic and the axonal sodium channels.

We next tested whether the asymmetric voltage attenuation is indeed a key component underlying the generation of spikelets through somato-dendritic input. For this, we turned to a model consisting of a dendrite, a soma, and an axon that all expressed active conductances (Fig 3A; see Methods for details). Similarly to the detailed compartmental model in Fig 1, the sodium channels at the distal AIS and in the axon were set to activate and inactivate at more hyperpolarized voltages than the sodium channels in the dendrite, the soma, and the proximal AIS [16, 21]. However, the model in Fig 3 is much simpler than the complex model in Fig 1, which enabled us to explore its parameter space.

To study the response of the model neuron with a simple stimulus, we applied rectangular current pulses (50 ms) to the soma for a range of input strengths. When an AP at the AIS was evoked, the corresponding somatic maximum response amplitude was recorded and plotted in a continuous color code (Fig 3B–3H). However, the somatic response amplitudes typically appeared in three well-separated clusters (examples in Fig 3B and S2 Fig B): (i) Spikelets (yellow) resulted from the weakest inputs that generated APs at the AIS but failed to evoke a somatic AP. (ii) The sh-APs (red) were evoked by larger somatic inputs and resulted from APs at the AIS that evoked a somatic AP. The shoulders of the sh-APs matched the spikelet waveform (see phase plots in Fig 3B, right). (iii) Finally, strong enough inputs could lead to full-blown APs (fb-APs; orange), which did not display a shoulder. The fb-APs resulted from AP initiation at the soma before or concurrent with AP initiation at the AIS. Consequently, fb-APs lacked the rapid onset (“kink”) typical for spikelets and sh-APs (Fig 3B, right) and the fb-AP amplitudes (from maximum curvature to maximum voltage) appeared smaller than the amplitudes of sh-APs because the maximum curvature occurred at higher voltages (Fig 3B, right). So similarly to the detailed model from Fig 1, input amplitude determined whether a spikelet or an AP was generated at the soma (see also S2 Fig): passive somatic depolarization from the input current added up to the somatic depolarization due to the AP propagated from the AIS, and if it reached the (fixed) somatic threshold, an AP was generated at the soma. Otherwise, a somatic spikelet appeared.

To quantify how the somatic response type (spikelet, sh-AP, or fb-AP) depends on the somatic stimulus amplitude and the model parameters, we performed extensive numerical simulations of the active model with reduced morphology (Fig 3C–3H). These simulations indicated that the occurrence of spikelets required a certain degree of electrotonic separation between the soma and the AIS (Fig 3C and 3D) to allow for sufficient attenuation from axon to soma, as was suggested by the analytical results from the passive-membrane model (see Fig 2B–2D). Furthermore, spikelet generation needed a high enough somatic input capacitance (Fig 3E), in agreement with the analytical result that membrane capacitance was the primary current sink for APs propagating from AIS to soma (Fig 2F and 2G). Also as predicted, spikelet activity depended only weakly on the membrane resistance in a range that is plausible for pyramidal neurons (Fig 3F).

Besides the passive membrane characteristics, also active properties of sodium channels were fundamental to the generation of somatic spikelets (Fig 3G and 3H). Lowering somato-dendritic sodium channel densities increased the somatic firing threshold and thereby promoted spikelet occurrence (Fig 3G). This result is in agreement with the reduced sodium channel densities boosting spikelet generation in the multi-compartment model in Fig 1. Another way to increase the firing-threshold difference between the soma and the AIS and thereby facilitate spikelet occurrence was to introduce a voltage shift in the activation function between the somato-dendritic and the axonal sodium channels (Fig 3H). The voltage shift had to be large enough such that an AP initiated at the AIS did not reach the voltage threshold in the soma.

In summary, the simulation results of the active model with reduced morphology confirm that spikelets can be evoked through sufficiently small somatic input. In addition to strong and asymmetric voltage attenuation, the generation of spikelets requires a substantially lower AP threshold in the AIS compared to the soma.

### Orthodromic versus antidromic spikelets

Spikelets of axonal origin can be evoked with distal axonal stimulation when the antidromically propagating AP does not suffice to cross the somatic spiking threshold. Such antidromic spikelets could also result from axo-axonic coupling by gap junctions [8]. Since the antidromic spikelets have different functional consequences than the orthodromic spikelets shown in Figs 1 and 3, it is important to be able to distinguish the two phenomena.

**Fig 4:**
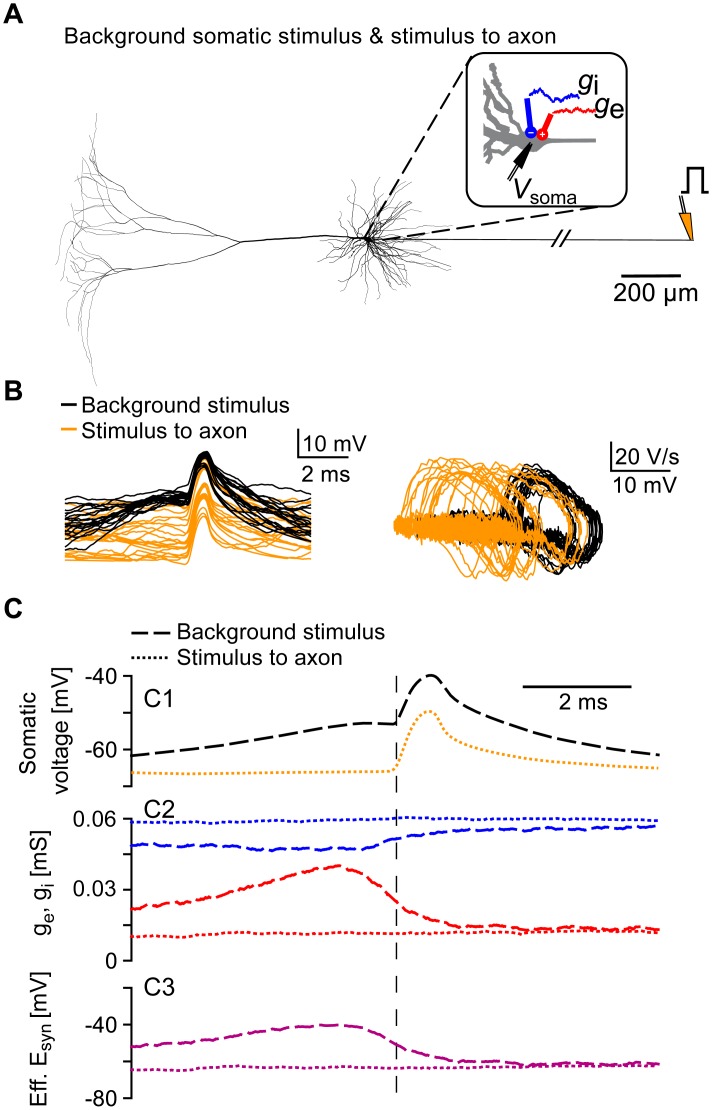
Orthodromic and antidromic spikelets in the biophysically complex model. A: Neuron model with fluctuating somatic inputs as in Fig 1 (red: excitatory, blue: inhibitory). Additionally, the model cell was stimulated every 500 ms with a short current pulse at the distal axon (orange, see Methods). B: Left: example somatic spikelets; shown are 20 orthodromic (black, evoked with somatic inputs) and 20 antidromic spikelets (orange, evoked with distal axonal inputs). Right: phase plots of the spikelets depicted in the left panel. C: Spikelet-triggered averages for all orthodromic spikelets (*N* = 66, dashed lines) and all antidromic spikelets (*N* = 194, dotted lines) generated within 100 s of simulation. C1: Mean orthodromic (dashed black) and antidromic (dotted orange) spikelet, aligned to the voltage-threshold crossing at the AIS (as in Fig 1H). C2: Mean excitatory (red) and inhibitory (blue) conductances for orthodromic (dashed lines) and antidromic (dotted lines) spikelets. C3: Mean effective synaptic reversal potentials (as in Fig 1H) for the orthodromic (dashed line) and antidromic (dotted line) spikelets.

To compare the properties of orthodromic and antidromic spikelets, the detailed model neuron with fluctuating somatic inputs from Fig 1 was additionally stimulated with brief current pulses to the distal axon (Fig 4A), which evoked axonal APs propagating antidromically towards the soma. The resulting spikelets were classified as antidromic (evoked with the distal axonal stimulus) and orthodromic (evoked with the somatic stimulus). Classification was based on the relative timing of the AP occurring at the distal AIS and in the axon (Fig 4B; see Methods). The two spikelet types were similar in shape and amplitude (Fig 4B and 4C), but the averaged antidromic spikelet displayed a more hyperpolarized somatic threshold and started abruptly from the baseline without a preceding depolarization (Fig 4C1), which is also typical for experimentally recorded antidromic APs [15]. For the antidromic spikelets in our computational model, the somatic excitatory and inhibitory conductances as well as the effective synaptic reversal potential did not show any modulation, which is in line with its distal axonal origin and its independence from somatic activity (Fig 4C2 and 4C3).

### Spikelets evoked by dendritic inputs

**Fig 5:**
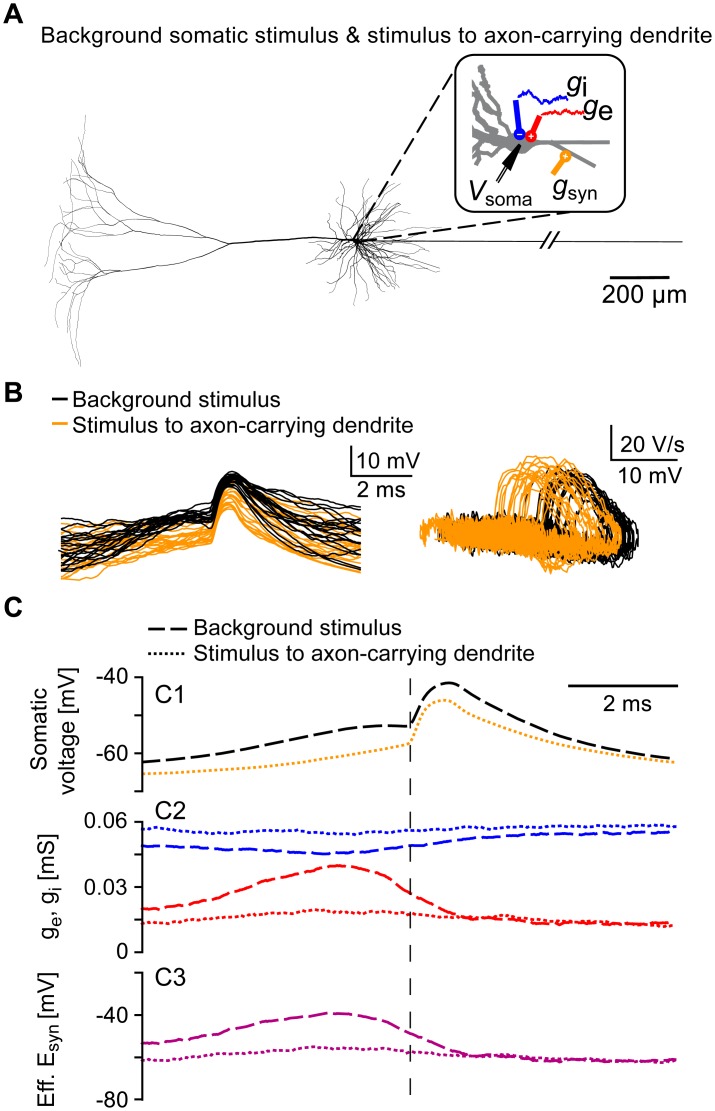
Orthodromic and antidromic-like spikelets in a model cell with the axon attached to a basal dendrite. A: Neuron model with fluctuating somatic inputs as in Fig 1 (red: excitatory, blue: inhibitory), except that the axon is attached to a basal dendrite. Additionally, the model cell was stimulated every 500 ms with a synaptic conductance *g*_syn_ located at the axon-carrying basal dendrite, distally to the AIS-connecting site (orange, see Methods). B: Left: example somatic spikelets; shown are 20 orthodromic (black, evoked with somatic inputs) and 20 antidromic-like spikelets(orange, evoked with dendritic input). Right: phase plots of the spikelets shown in the left panel. C: Spikelet-triggered averages for all orthodromic spikelets (*N* = 137, dashed lines) and all antidromic-like spikelets (*N* = 100, dotted lines) generated within 100 s of simulation. C1: Mean orthodromic (dashed black) and antidromic-like (dotted orange) spikelet, aligned to the voltage-threshold crossing at the AIS (as in Fig 1H). C2: Mean excitatory (red) and inhibitory (blue) conductances for orthodromic (dashed lines) and antidromic-like (dotted lines) spikelets. C3: Mean effective synaptic reversal potentials (as in Fig 1H) for the orthodromic (dashed line) and antidromic-like (dotted line) spikelets.

Although the physiological occurrence of antidromic spikelets is disputed [22], we hypothesized that spikelets with similar properties can occur in pyramidal cells when the axon is attached to a dendrite instead of the soma [23]. To simulate this scenario, we adapted the morphology of the detailed model cell used in Figs 1 and 4 (Fig 5; see Methods), and excitatory postsynaptic conductances (EPSGs) were delivered to the axon-carrying dendrite, additionally to the somatic fluctuating inputs (Fig 5A). The resulting spikelets (Fig 5B) were classified according to the relative timing of the spikelet and the EPSG (see Methods). Both types of spikelets had comparable shapes and phase plots (Fig 5B). Spikelets evoked with stimuli to the axon-carrying dendrite exhibited a hyperpolarized average onset; nevertheless, some depolarization preceding these spikelets was visible in the somatic traces because the underlying input was located close enough to the soma (≈ 25 *μ*m). However, spikelets evoked with stimuli to the axon-carrying dendrite were basically independent of somatic synaptic conductances (Fig 5C), and these spikelets are therefore reminiscent of the antidromic spikelets described in Fig 4.

**Fig 6:**
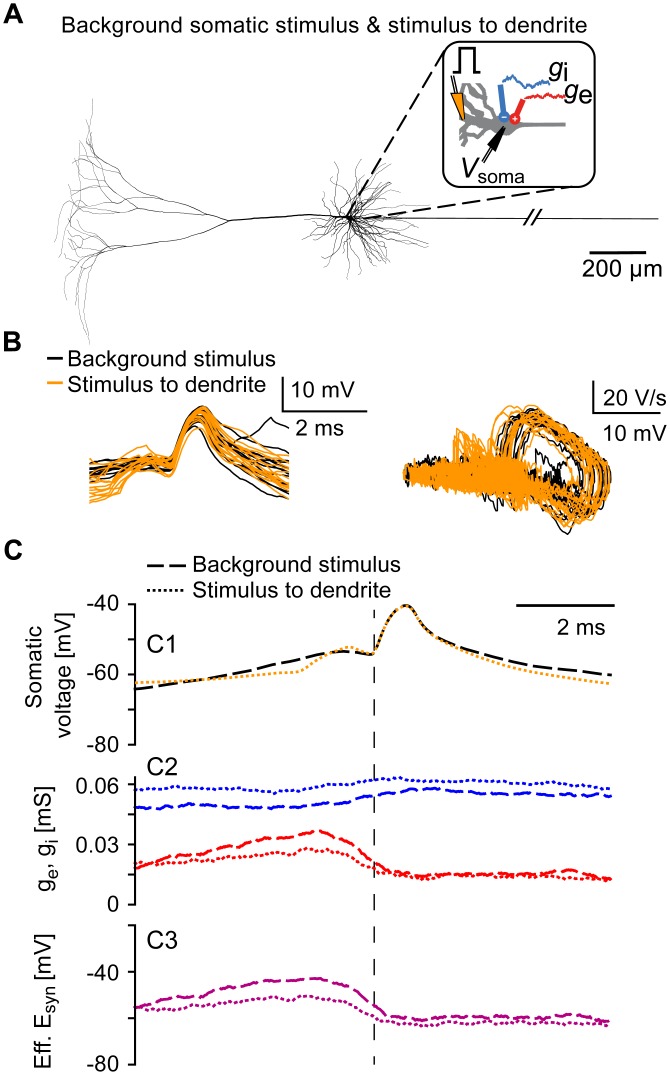
Orthodromic spikelets evoked with somatic background inputs and dendritic current stimuli. A: Neuron model with fluctuating somatic inputs as in Fig 1 (red: excitatory, blue: inhibitory). Additionally, the model cell was stimulated every 20 ms with a brief current pulse at the proximal apical dendrite (orange, see Methods). B: Left: example somatic spikelets; shown are 15 spikelets evoked with the dendritic stimulus (orange) and 15 spikelets evoked with the somatic background stimulus (black). Right: phase plots of the depicted spikelets. C: Spikelet-triggered averages for all spikelets evoked with the somatic background stimulus (*N* = 41, dashed lines) and all spikelets triggered by the dendritic input (*N* = 43, dotted lines) generated within 200 s of simulation, see Methods. C1: Mean spikelets evoked with the somatic background stimulus (black dashed line) and with the dendritic stimulus (orange dotted line), aligned to the voltage-threshold crossing at the AIS (as in Fig 1H). C2: Mean excitatory (red) and inhibitory (blue) conductances for spikelets evoked with the somatic background stimulus (dashed lines) and for spikelets evoked with the dendritic stimulus (dotted lines). C3: Mean effective synaptic reversal potentials (as in Fig 1H) for spikelets evoked with the somatic background stimulus (dashed line) and with the dendritic stimulus (dotted line).

Alternatively, when the model presented in Fig 1 was additionally stimulated with brief current pulses at the proximal apical dendrite, the thresholds and waveforms of spikelets resulting from the dendritic stimulus were virtually identical to spikelets triggered by the fluctuating background stimulus applied to the soma (Fig 6). The average background conductances (Fig 6C2) and the effective synaptic drive (Fig 6C3) were less modulated for the dendritically evoked spikelets than for the spikelets evoked with the background stimulus. The number of dendritically evoked spikelets was substantially smaller than for inputs located at the distal axon or at the axon-attached dendrite because of an interplay between the dendritic and somatic stimulus in spikelet generation: The dendritic stimulus added to the background somatic input and triggered spikelets if the soma had the right level of depolarization. If the soma was too depolarized at the time point when the dendritic stimulus arrives, somatic APs were evoked; if the soma was too hyperpolarized, the compound input did not suffice to trigger an AP at the AIS.

**Fig 7:**
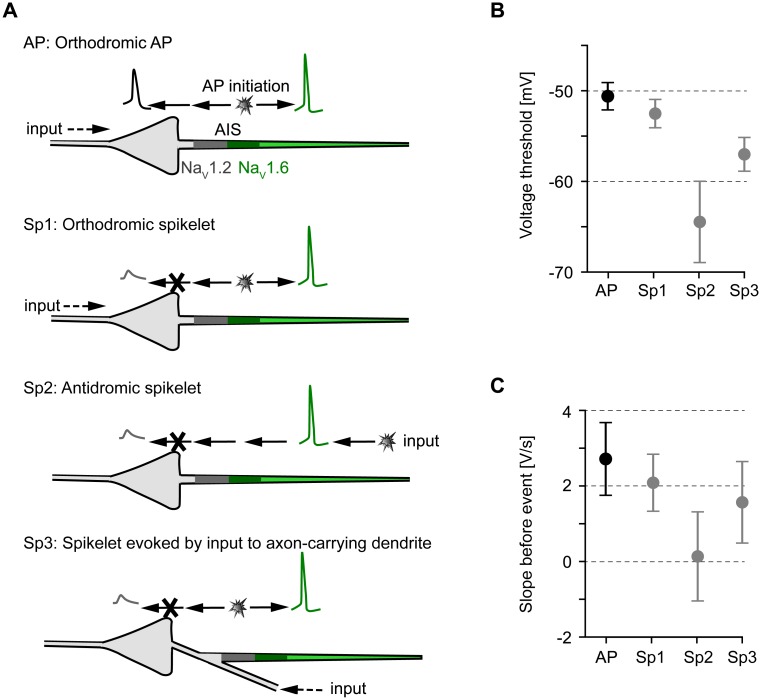
Mechanisms of spikelet generation in pyramidal neurons. A: Sketch of the pyramidal-cell neuron model. The axon initial segment (AIS) can be divided in the proximal part (dark gray), where high-threshold Na_V_1.2 channels accumulate, and the distal part, where low-threshold Na_V_1.6 channels accumulate (dark green). High-threshold Na_V_1.2 channels are present at lower densities throughout the soma and dendrites (light gray). Low-threshold Na_V_1.6 channels are located throughout the axon (light green), but at lower densities than in the distal AIS (see Methods). We distinguish four different scenarios (AP, Sp1, Sp2, Sp3), which are described in detail in what follows. AP: Strong enough somato-dendritic inputs initiate an AP at the distal AIS (dark green). The AP then propagates down the axon and back to the soma and into the dendrites. Sp1: Weaker and briefer somato-dendritic inputs give rise to somatic spikelets if the AP initiated at the AIS fails to trigger a somatic AP. However, the axonal AP propagation to the postsynaptic targets remains unaffected. Sp2: Antidromic spikelets occur when an AP initiated in the distal axon propagates to the soma, but does not suffice to evoke a somatic AP. Sp3: In neurons with the axon connected to a basal dendrite, spikelets can also be evoked by inputs to the axon-carrying dendrite. These inputs can evoke an AP at the AIS without passing the soma first. The evoked AP, in turn, propagates down the axon but might fail to trigger a somato-dendritic spike, so a somatic spikelet appears. B: Mean somatic voltage threshold for the four scenarios illustrated in A: orthodromic APs (AP, *N* = 579), orthodromic spikelets (Sp1, *N* = 63), antidromic spikelets (Sp2, *N* = 194), and spikelets evoked by inputs to the axon-carrying dendrite (Sp3, *N* = 100). Error bars mark standard deviation. C: Mean somatic voltage slope in the 5-ms interval before the event, for the four scenarios illustrated in A.

To summarize our results, spikelets can be generated within a single pyramidal neuron in three ways (Fig 7A, Sp1–Sp3). Each type of spikelet has characteristic features, which may allow to infer the origin of spikelets in experimental somatic voltage traces. Two key distinguishing features of spikelets are the somatic voltage threshold (Fig 7B) and the slope of the voltage a few milliseconds before the threshold is reached (Fig 7C). As a reference we consider the orthodromic APs, which exhibit the highest somatic firing threshold and are preceded by the steepest depolarization compared to the three types of spikelets: Orthodromic spikelets (Sp1) show a slightly smaller threshold and are preceded by a less steep depolarization, consistent with the finding that they required weaker inputs than APs. Antidromic spikelets (Sp2), which were evoked in our simulations with distal axonal stimulation, are characterized by the lowest thresholds and the highest somatic threshold variability. They arise abruptly at the soma: the averaged voltage trace shows no preceding depolarization. Finally, spikelets evoked by inputs to the axon-carrying dendrite (Sp3) lie somewhere in between the orthodromic and antidromic spikelets, regarding the average somatic threshold and the preceding depolarization; their orthodromic-like versus antidromic-like appearance depends on the electrotonic separation of the soma and the axon-carrying dendrite.

## Discussion

Action potentials are the basis of neural function, yet some of their fundamental features are still not well understood, as highlighted by the recent focus on the rapidness of the AP onset [19, 24, 25]. It is generally assumed that an AP initiated in the AIS of a pyramidal neuron always leads to an AP in the soma. We argue here that this view needs to be corrected. Under certain conditions, APs initiated in the AIS by somato-dendritic inputs fail to fully activate the soma and appear there as spikelets.

In simulations we showed that spikelets can result from APs that were evoked at the AIS with somato-dendritic inputs and propagated down the axon, but that did not trigger a somato-dendritic AP. This AP failure occurred for a sufficiently large difference in spiking thresholds between the soma and the AIS, together with a strong impedance mismatch (causing asymmetric voltage attenuation) and some degree of electrotonic separation between the soma and the AIS. In this way, a weak depolarizing input could pass through the soma and initiate an AP at the AIS, which, in turn, was not able to depolarize the soma to the firing threshold. Thus, a spikelet appeared at the soma instead of an AP.

This mechanism reproduced several key features of spikelets reported in the experimental literature [2, 4, 5]: the fast dynamics and rapid onset of spikelets as well as the match between the spikelet waveform and the shoulder of a sh-AP. This single-cell mechanism is also in line with the observation that APs and spikelets recorded in a single hippocampal place cell exhibit virtually identical place fields [2]. In contrast, in the electrotonic-coupling (gap junction) scenario of pairs of pyramidal cells [8–10], the place fields of spikelets and APs measured in a single cell are expected to differ due to lack of topography in hippocampus [26]. We found that the fast dynamics and amplitudes of spikelets observed in pyramidal neurons can be compatible with gap junction coupling only if the somato-dendritic gap junctions are very strong and located at proximal sites (S3 Fig).

In previous experimental studies, spikelets could be evoked with dendritic stimulation or dendritic EPSPs [4, 6], which led the authors to conclude that somatic spikelets arise from dendritic spikes. However, our modelling results suggest that although spikelets can be evoked with somato-dendritic inputs, they rather originate in the axon. Depending on the state of the proximal axonal sodium channels, the AP is initiated either in the AIS, as we considered in this study, or further down the axon. Consistently, a recent experimental study demonstrated an axonal origin of spikelets occurring during dendritic plateau-driven complex spiking in CA1 pyramidal neurons [27]. Also in other central neurons, spikelets occurring during somatic bursts can originate in the axon, for example, in inferior olivary neurons [28] and in cerebellar Purkinje neurons [29].

Antidromic spikelets also result from axonal APs, but these are evoked by distal axonal inputs [30] or by APs propagating through putative axo-axonal gap junctions [8]. Compared to the orthodromic spikelets, antidromic spikelets are characterized by hyperpolarized thresholds and they arise abruptly without a preceding depolarization (Fig 4C1). However, the best experimental distinguishing criterion is the fact that, because of their distal origin, they survive moderate levels of somatic hyperpolarization, as has been demonstrated, for example, in layer V pyramidal neurons *in vitro* [16]. Orthodromic spikelets do not occur when the somatically injected hyperpolarizing current is larger than the synaptic driving current measured at the soma, since the synaptic depolarizing input has to pass through the soma to trigger an AP at the AIS. In contrast, antidromic spikelets can be evoked even when the synaptic driving current is somewhat smaller than the somatically injected hyperpolarizing current. Spikelets evoked by inputs to the axon-carrying dendrite (Fig 5) would also be abolished by a certain level of somatic hyperpolarization, because of the relatively small electrotonic distance between the soma and the axon origin [23]. Consistent with an orthodromic origin of spikelets is the experimental observation that spikelets are suppressed by hyperpolarizing somatic current injections, leading to the conclusion that spikelets “are not generated far from the soma” [4].

Our proposed spikelet hypothesis relies on AP initiation at the AIS. Indeed, APs in hippocampal [31] and neocortical pyramidal neurons [16, 32] are typically initiated in the distal portion of the AIS, about 20 − 40 *μ*m away from the axon hillock. This site is preferred for AP initiation because of its decreased capacitive load from the soma [33] and increased sodium channel density, especially of the Na_V_1.6 channel subtype [34], which activates at more hyperpolarized membrane potentials than the somatic sodium channel subtype Na_V_1.2 [21]. However, it is still disputed whether the axonal sodium channel density is substantially higher (up to 50-times higher, [35]) than the somatic sodium channel density or whether the axonal and somatic sodium channels have similar densities [21, 36].

The model neuron used in Figs 1 and 4–6 is characterized by a high ratio between the axonal and somatic sodium channel densities (up to a factor 40, [16]), which contributes to the large threshold difference between the axon and the soma, thus favoring spikelet generation. The question then arises how spikelet generation is affected when the sodium channel density ratio is smaller. The model used in Fig 3 employed a much smaller density ratio of 5 between the soma and the distal AIS (0.02 and 0.1 S/cm^2^, respectively). Fig 3G illustrates that spikelets occurred when the somatic sodium channel density was less than half the value at the distal AIS (i.e., < 0.05 S/cm^2^). *In vivo*, a fraction of somatic sodium channels is inactivated due to ongoing activity, which decreases the effective sodium channel density and promotes spikelet occurrence. However, the range of density ratios that support spikelet generation is not absolute, but depends on other parameters influencing somatic voltage threshold, like the voltage shift between the activation of somatic and axonal sodium channels (Fig 3H).

In the present study, we used the standard sodium channel models that were fitted to neocortical (Figs 1 and 4–6, [16]) and hippocampal (Fig 3, [37]) pyramidal neurons. However, the dynamics of these model channels is slow compared to what has been found in more recent experiments [38, 39]. Interestingly, simulations by Fleidervish et al. demonstrated that the faster, more realistic, sodium channel activation generated larger axo-somatic delays and larger voltage gradients than the classic, slower, sodium channel models [36]. As this axo-somatic gradient is vital for spikelet generation, we expect faster Na-channel gating to support spikelet generation.

Experimental recordings featuring spikelets typically contain two types of APs: shoulder-APs with an initial slower phase corresponding to the spikelet, and full-blown APs, characterized by a single rising phase without a shoulder [2]. The shoulder of sh-APs is considered to result from the AP evoked at the AIS (e.g., [19]). Then, the question about the origin of fb-APs arises. In our detailed compartmental model (Fig 1), all APs are evoked at the AIS and exhibit a shoulder. In the simple model shown in Fig 3, fb-APs can be generated with strong stimuli and for large electrotonic distances between the soma and the AIS, which allows somatic AP initiation to precede or co-occur with AP initiation at the AIS. However, unlike experimentally recorded fb-APs, they arise smoothly from the subthreshold depolarization and do not exhibit a rapid onset that is present in simulated and experimentally recorded spikelets and sh-APs. According to the “compartmentalization hypothesis of AP initiation” [25], the AP onset rapidness is caused by axonal AP initiation. This suggests that experimentally recorded fb-APs with rapid onset are not generated at the soma. Consistently, somatic AP initiation due to serotonin inhibition of AIS channels can result in gradually rising APs without a rapid onset [40]. Therefore, we hypothesize that fb-APs are either generated at the AIS and the shoulder is “masked” by fast somato-dendritic activation or they are initiated in the apical dendrites and no shoulder is visible because of the smooth morphologic transition between the primary apical dendrite and the soma.

An intriguing issue concerns the rare observation of spikelets *in vitro*. Our analyses suggest that pyramidal neurons are positioned at the edge of a regime that allows spikelet generation. In the complex model from [16] used in Figs 1 and 4–6 for example, a modest decrease in sodium channel density strongly increased spikelet occurrence. One reason for such a decrease in functional sodium channel availability might be slow sodium channel inactivation [41]. *In vitro*, there is less slow sodium channel inactivation: a larger fraction of sodium channels might be available for spiking due to a lower average membrane potential and a lower firing activity, which keeps the fraction of inactivated sodium channels low. Additionally, sodium channel availability is regulated by various neuromodulators, acting via activity-dependent phosphorylation [42]. This might be especially relevant *in vivo*, where a variety of homeostatic mechanisms are expected to maintain spiking activity in neural circuits [43]. In our models, fast sodium channel inactivation was not a main factor influencing spikelet generation (S1 and S2 Figs). It cannot be ruled out, however, that fast sodium inactivation does play a significant role in real neurons under certain *in vivo* conditions.

Another important factor for spikelet generation is the somato-dendritic current sink, which is reduced in brain slices because of “dendritic pruning”, i.e., dendritic processes cut by the slicing procedure [44]. The typical thickness of slices is a few hundred microns (e.g., 300 *μ*m, [8, 16]), which roughly matches the spatial extent of a pyramidal neuron’s dendritic tree (e.g., [45]). For patch-clamp recordings, cells close to the slice surface are preferentially used, which is where one expects significant damage to proximal dendrites [44]. A pyramidal cell’s input capacitance is in the range of hundreds of picofarads [46], and considerable changes of this value are predicted to strongly affect spikelet occurrence (Fig 3E). In contrast, an artificial capacitance increase of about 4—10 pF by an uncompensated patch electrode [47] is small compared to a pyramidal cell’s input capacitance and, thus, should not influence spikelet incidence significantly.

The presented hypothesis predicts that all-or-none somatic spikelets in pyramidal neurons are associated with APs at the AIS or further down in the axon [27]. This mechanism could be tested experimentally with simultaneous recordings of the somatic and axonal membrane voltages, which, however, might be difficult *in vivo*. An alternative would be to establish a reliable spikelet model *in vitro*. We propose to recreate *in vitro* a state of a pyramidal cell that retains the *in vivo* properties of sodium channels, for example by prolonged stimulation with fluctuating inputs and/or application of relevant neurotransmitters and neuromodulators naturally present in the cerebrospinal fluid *in vivo* [48]. Additionally, it might be necessary to record from neurons located in the middle of a slice, to minimize the dendritic loss and the resulting decrease in the somato-dendritic current sink.

Interestingly, unlike in mammalian cells, spikelets are easily evoked in turtle pyramidal neurons *in vitro* with weak somatic or dendritic stimuli [49, 50]. The amplitudes and waveforms of these spikelets closely resemble those in mammalian pyramidal neurons. Dual somatic and axonal recordings suggested an axonal origin of these spikelets [50]. We hypothesize that there might be two important differences between turtle and mammalian neurons that support *in vitro* spikelet firing in turtles. First, the slower and wider APs in turtles suggest that the effective (peri-)somatic sodium channel densities might be smaller in turtle than in mammalian pyramidal neurons. Second, the somata of turtle neurons are substantially larger than the somata of mammalian neurons, and most of the dendrites are single branches extending from the soma [50]. This might result in an increased capacitive somato-dendritic current sink and augment the impedance mismatch between the axon and the soma.

The spikelets we described here are APs that propagate forward down the axon but not backward into the soma and the dendrites. What could be a functional role of such “output-only APs”? From an energetic point of view, spikelet firing saves energy since it avoids activation of sodium currents in the soma and the dendritic tree. Output-only APs thus minimize their contribution to activity-dependent metabolism [51, 52]. Moreover, spikelets might be a means of reading out the result of neuronal computations without triggering dendritic plasticity through backpropagating APs [53]. Hence, spikelets potentially represent a mode of operation that is functionally highly relevant.

To further unravel the role spikelets may play in neural computations, more theoretical and experimental studies are needed. Developing a CA1 pyramidal neuron model with a realistic AIS composition incorporating state-of-the-art sodium channel models is vital for a quantitative study of spikelet generation and properties, as the prevailing experimental work on spikelets has been carried out in these neurons. In order to construct such a model, further experimental studies of AIS composition and function in CA1 pyramidal neurons are necessary. Future studies could also address the putative role of axo-axonic synapses in spikelet generation, which provide powerful inhibition at the proximal AIS that can prevent antidromically evoked APs from invading the soma [12]. It would be important to see whether these synapses can control the propagation of orthodromically initiated APs and give rise to somatic spikelets, given the small distances between the soma and the distal AIS and the requirement for precise timing of inhibition: Too early inhibition would shunt the subthreshold depolarization and prevent AP initiation in the first place, whereas too late inhibition would be ineffective to stop the propagating AP (see also [54]). Also the influence of sodium channel neuromodulation on spikelet occurrence [42] and generation of full-blown APs in cells exhibiting spikelets are important topics for our understanding of spikelets in pyramidal neurons. This knowledge should allow to assess the computational consequences of spikelet firing at the single-cell and network level.

## Methods

### Detailed compartmental model

For the results in Figs 1, 4, 5 and 6 we used a previously published detailed model of a reconstructed layer V pyramidal neuron [16, ModelDB accession number 123897], implemented in NEURON [55]. Compared to the original model, we made two modifications. First, a small geometrical discontinuity at the AIS was corrected. In the original model, the AIS tapers from 1.7 *μ*m to 1.22 *μ*m. However, the diameter at the end of the axon hillock, i.e., at the hillock-AIS boundary, is 1.3 *μ*m. We removed this sudden jump in the diameter so that the diameters at the end of the axon hillock and at the beginning of the AIS are equal at a value of 1.3 *μ*m (then tapering smoothly to 1.22 *μ*m, at the end of AIS). Second, the density of the Na_V_1.2 subtype was decreased in soma, axon hillock, and AIS to 80%, and in dendrites to 60% of the original values. These changes only weakly influenced the AP properties and firing patterns (Table 1). The largest effects were observed for spikelet frequency and maximum AP slope. The decrease in maximum AP slope was desired, as it reflects the smaller AP slopes reported *in vivo*. Overall, the properties of APs generated in this model (Table 1) fit well into the range reported for pyramidal neurons in the experimental literature [2, 5, 24, 32, 56].

**Table 1:** Comparison of AP- and firing properties in the original model and the adapted model used in Fig 1.

Model properties	Original model^1^	Original gNa + corrected diameter	Adapted model^2^
AP threshold (kink)* [mV]	-49.87	-50.01	-50.31
AP amplitude* [mV]	92.54	92.97	85.99
Width of AP at half amplitude* [ms]	0.71	0.71	0.78
Max. AP dV/dt* [mV/ms]	349.53	359.22	261.82
AP firing rate^#^ [APs/s]	6.57	6.57	5.79
Spikelet firing rate^#^ [spikelets/s]	0	0.04	0.63
std(Vm@soma)^#^^∘^ [mV]	8.51	8.52	8.09

^1^ original model [16]: original Na channel densities (gNa) and diameter discontinuity at hillock—AIS boundary

^2^ adapted model used in Fig 1: reduced Na channel densities (gNa) and corrected diameter discontinuity as described in the Methods

* single APs evoked with somatic current pulses (1 nA for 10 ms)

^#^ 100 s simulation with stochastic synaptic conductances like in Fig 1

^∘^ standard deviation of somatic membrane voltage

The compartmental model cell was stimulated with two fluctuating synaptic point conductances placed at the soma [18] with the following parameters (values given in parentheses): reversal potential of the excitatory (*E*_*e*_ = 0 mV) and inhibitory (*E*_*i*_ = −75 mV) conductance, average excitatory (*g*_*e*0_ = 0.01 *μ*S) and inhibitory (*g*_*i*0_ = 0.0573 *μ*S) conductance, standard deviation of the excitatory (std_*e*_ = 0.014 *μ*S) and inhibitory (std_*i*_ = 0.02 *μ*S) conductance and time constant of the excitatory (*τ*_*e*_ = 2.728 ms) and inhibitory (*τ*_*i*_ = 10.49 ms) conductance. As a result, the somatic membrane voltage fluctuated with a standard deviation of 8.09 mV, producing a somatic AP firing rate of 5.79 s^−1^ and a spikelet firing rate of 0.63 s^−1^ (Fig 1).

The somatic APs and spikelets were detected using a voltage-threshold criterion at the AIS and at the soma (both − 10m*V*). For both types of events, the threshold at the AIS had to be crossed. If the threshold at the soma was crossed within a time window from 1 ms before to 5 ms after the AIS threshold crossing, such an event was classified as an AP. Otherwise, the event was a spikelet. We also used a double-threshold criterion for the somatic voltage derivative (dV/dt) to confirm that no event was missed by the above voltage-threshold criterion and that indeed all somatic APs and spikelets were associated with an AP at the AIS: events that crossed the first threshold (20 V/s), but not the second threshold (100 V/s) were classified as spikelets, whereas somatic APs had to cross both thresholds within 2 ms.

In Fig 1E, the APs were aligned in time to the point of crossing a somatic voltage threshold of -10 mV, whereas spikelets were aligned to the point of crossing a voltage threshold of -10 mV at the AIS. In Fig 1H, all events were aligned to the point of crossing the voltage threshold at the AIS to allow for a comparison of inputs between APs and spikelets. In Fig 1H, the effective synaptic reversal potential was calculated as (*g*_*e*_(*t*)*E*_*e*_ + *g*_*i*_(*t*)*Ei*)/(*g*_*e*_(*t*) + *g*_*i*_(*t*)), i.e., the excitatory and inhibitory reversal potentials weighted with the respective conductances.

In Fig 4, in addition to the somatic conductance inputs as in Fig 1, the model cell was also stimulated with brief current pulses (0.5 nA for 2 ms) delivered every 500 ms at the most distal axonal compartment. Somatic spikelets were classified as orthodromic (i.e., evoked with somatic inputs) or antidromic (i.e., evoked with distal axonal inputs) based on the relative timing of the AP at the distal AIS and in the axon. For orthodromic spikelets, the AP at the distal AIS preceded the AP in the axon; for antidromic spikelets, the AP at the distal AIS followed the AP in the axon.

In Fig 5, the morphology of the model cell was altered: the axon hillock was omitted and the AIS was attached to a basal dendrite (“dendrite3[2](0.5)”) 20.5 *μ*m away from the soma. In addition to the somatic conductance inputs as in Fig 1, an EPSG (*τ*_rise_ = 0.5 ms, *τ*_decay_ = 2 ms, peak conductance = 0.02 *μ*S, *E*_syn_ = 0 mV) was delivered every 500 ms to the axon-carrying dendrite, distally to the AIS-connecting site (“dendrite3[3](0.1)”). Spikelets evoked with dendritic EPSGs were distinguished from the orthodromic spikelets (evoked with somatic inputs) as spikelets occurring within a 2 ms window after the dendritic EPSG.

In Fig 6, in addition to the somatic conductance inputs as in Fig 1, the model cell was also stimulated with a brief current pulse (2 nA for 1 ms) delivered every 20 ms at the proximal apical dendrite (“dendrite11[2](0)”) 47 *μ*m away from soma. In 200 s of simulation, 2,106 somatic APs and 91 somatic spikelets were generated. We classified the spikelets as evoked with the dendritic input if the somatic spikelet was evoked within 2 ms from dendritic stimulus onset (*N* = 43); if the spikelet occurred 10 ms or later after the onset of the dendritic stimulus, the spikelet was classified as triggered by the somatic background stimulus (*N* = 41).

In S3 Fig, we simulated two identical cells (as in Fig 1) coupled by a gap junction. The gap junction was modelled as an ohmic resistor, allowing to transmit voltage changes between the coupled cells [57]. In cell 1, an AP was evoked with a somatic current step (2 nA applied for 15 ms), and a spikelet was recorded in cell 2. The strength of the gap junction was varied between 22 and 82 MΩ in 5 MΩ steps (corresponding to gap junctional conductance of 12–45 nS). The gap junction was placed at the soma or at several positions along the main apical dendrite (at a distance of ≈ 8, 24, 47, 78, or 109 *μ*m from soma). The leak reversal and initial membrane voltages were set to -80 mV instead of the original leak reversal of -70 mV because otherwise the closest and strongest gap junctions could only generate an AP and not a spikelet in cell 2. The amplitude of spikelets was measured from the maximum of the 2nd derivative (the “kink”) to the maximum amplitude.

### Passive-membrane model of an axonal cable and a somato-dendritic compartment

We mathematically analyzed a model consisting of a semi-infinite cable with an RC-circuit as a boundary condition, representing the axon and the entire somato-dendritic compartment, respectively (Fig 2). The system is mathematically equivalent to the lumped-soma model introduced by Rall [58]. Our model describes the dynamics of the voltage *V* along the axon at distance *x* from the soma in response to current input at location *x* = *y* using the linear cable equation:

$$\lambda^{2}\frac{\delta^{2}}{\delta x^{2}}V\left(x,t\right)-\tau \frac{\delta }{\delta t}V\left(x,t\right)-V\left(x,t\right)=g\left(x,t\right)\;\;\text{for}\;\;x>0$$ (1)

where *τ* is the membrane time constant (in ms), λ is the axonal length constant (in cm), and *g*(*x*, *t*) is the input to the model. The boundary condition to include the somato-dendritic compartment at *x* = 0 is

$$\tau \frac{\delta }{\delta t}V\left(0,t\right)=\lambda \rho \frac{\delta }{\delta x}V\left(0,t\right)-V\left(0,t\right)$$ (2)

where the dimensionless parameter *ρ* denotes the ratio of the total somato-dendritic membrane resistance to the input resistance of the axon. The semi-infinite cable boundary condition is

$$\lim_{x\to \infty }V\left(x,t\right)=0.$$ (3)

For notational convenience we consider the resting potential in this linear system to be 0 mV. The parameters *τ*, λ, and *ρ* are determined by physiological parameters. Setting the specific membrane resistance *R*_*m*_ = 10^4^ Ω cm^2^, specific membrane capacitance *C*_*m*_ = 1 *μF*/cm^2^, axial resistivity *R*_*a*_ = 150 Ω cm, surface area of the somato-dendritic compartment *A*_*sd*_ = 2 ⋅ 10^−4^ cm^2^ and diameter of the axon *d*_*a*_ = 10^−4^ cm yields *τ* = *R*_*m*_*C*_*m*_ = 10 ms, $\lambda =\sqrt{\frac{R_{m}d_{a}}{4R_{a}}}=0.041\;\text{cm}$ and $\rho =\frac{\pi d_{a}^{3/2}}{2A_{sd}}=0.064$.

The purpose of the mathematical model was to compute the frequency-dependent attenuation of voltage signals between the axon and the somato-dendritic compartment. One approach is to use a complex-valued input current in the original partial differential equation and solve for the voltage responses of the axon and the somato-dendritic compartment. Here, we will instead proceed using a real-valued input current and use the Fourier transforms of the above partial differential equation and boundary conditions:

$$\lambda^{2}\frac{\delta^{2}}{\delta x^{2}}\hat{V}\left(x,\omega \right)-b{\left(\omega \right)}^{2}\hat{V}\left(x,\omega \right)=\hat{g}\left(x,\omega \right)\;\;\text{for}\;\;x>0$$ (4)

with the boundary conditions

$$\frac{\delta }{\delta x}\hat{V}\left(0,\omega \right)-\frac{b{\left(\omega \right)}^{2}}{\lambda \rho }\hat{V}\left(0,\omega \right)=0$$ (5)

and

$$\lim_{x\to \infty }\hat{V}\left(x,\omega \right)=0,$$ (6)

where $\hat{V}\left(x,\omega \right)$ and $\hat{g}\left(x,\omega \right)$ are the Fourier transforms of *V*(*x*, *t*) and *g*(*x*, *t*), respectively, *ω* = 2*πf* with frequency *f* (in Hertz), and *b*(*ω*)^2^ = 1 + *iωτ*. We next calculated the voltage response of the model to the real-valued sinusoidal input current at location *x* = *y*:

$$g\left(x,t\right)=\frac{R_{m}}{\pi d_{a}}I_{0}\cos\left(\omega_{0}t\right)\delta \left(x-y\right)$$ (7)

with radial frequency *ω* = *ω*_0_ ≥ 0 and amplitude *I*_0_. The Fourier transform of the input term is

$$\hat{g}\left(x,\omega \right)=\frac{R_{m}}{\pi d_{a}}I_{0}\;\delta \left(\omega -\omega_{0}\right)\;\delta \left(x-y\right),$$ (8)

where we neglected the negative-frequency terms. We then solved the above second-order, nonhomogeneous ODE by first considering solutions of the form ${\hat{V}}_{h}\left(x,\omega \right)=c_{1}\exp\left(-b\left(\omega \right)\;x/\lambda \right)+c_{2}\exp\left(b\left(\omega \right)\;x/\lambda \right)$ for the homogeneous version of the ODE and use this to find a particular solution ${\hat{V}}_{nh}\left(x,\omega \right)$ for the nonhomogeneous ODE; subsequently the constants *c*_1_ and *c*_2_ were determined by considering the boundary conditions [59, section 6.2]. The sinusoidal voltage response at location 0 ≤ *x* ≤ *y* is

$$\hat{V}\left(x,\omega_{0}\right)=\frac{I_{0}R_{\infty }}{b_{0}}\;\left(\frac{\rho \cosh\left(b_{0}\;x/\lambda \right)+b_{0}\sinh\left(b_{0}\;x/\lambda \right)}{\left(b_{0}+\rho \right)\exp\left(b_{0}\;y/\lambda \right)}\right),$$ (9)

and for *x* ≥ *y* it is

$$\hat{V}\left(x,\omega_{0}\right)=\frac{I_{0}R_{\infty }}{b_{0}}\;\left(\frac{\rho \cosh\left(b_{0}\;x/\lambda \right)+b_{0}\sinh\left(b_{0}\;x/\lambda \right)}{\left(b_{0}+\rho \right)\exp\left(b_{0}\;y/\lambda \right)}-\sinh\left(b_{0}\;\left(x-y\right)/\lambda \right)\right)$$ (10)

where *b*_0_ = *b*(*ω*_0_) is the principal square root (i.e., with positive real part) of $\sqrt{1+i\omega_{0}\tau }$ and $R_{\infty }=\frac{2}{\pi }\;d_{a}^{-3/2}\sqrt{R_{m}R_{a}}$ is the input resistance of a semi-infinite cable. The steady-state voltage attenuation from axon to soma is then given by the ratio of the voltage response amplitude at the axonal injection site to the somatic voltage response amplitude:

$$A_{axon\to soma}\left(y,\omega_{0}\right)=\left|\frac{\hat{V}\left(y,\omega_{0}\right)}{\hat{V}\left(0,\omega_{0}\right)}\right|=\left|\cosh\left(b_{0}\;y/\lambda \right)+\frac{b_{0}}{\rho }\sinh\left(b_{0}\;y/\lambda \right)\right|,$$ (11)

where |*z*| denotes the absolute value of the complex number *z*. Similarly, the frequency-dependent voltage attenuation from soma to axon for a somatic input (i.e., *y* = 0 and *x* ≥ *y*) can be computed, which is equal to the attenuation in an (semi-) infinite cable:

$$A_{soma\to axon}\left(x,\omega_{0}\right)=\left|\frac{\hat{V}\left(0,\omega_{0}\right)}{\hat{V}\left(x,\omega_{0}\right)}\right|=\left|\exp\left(b_{0}x/\lambda \right)\right|.$$ (12)

In Fig 2B–2G, the natural logarithm of the attenuation was plotted. The axonal stimulation/recording site was *y* = 50 *μ*m away from the soma (except in Fig 2B where it was varied). The passive-membrane model was also simulated numerically with the NEURON module embedded in Python [60] to compare the antidromic (axon-to-soma) attenuation of pure sine waves with the attenuation of an AP waveform. Here, identical parameters were used as in the analytical calculations (see above). The axon length was set to 2 mm, corresponding to an electrotonic length of 4.9 λ. The AP waveform was delivered via a voltage clamp at a 1 *μ*m long axonal compartment located 50 *μ*m away from the soma. We used an AP waveform recorded at the AIS of the detailed model (Fig 1D, middle). The input capacitance in Fig 2G was calculated from a small, prolonged voltage-clamp step by dividing the integrated transient charge by the voltage-clamp step size [61].

### Active model with reduced morphology

Results presented in Fig 3 used an active compartmental model of a simplified neuron morphology. The model consisted of a dendritic cable (length × diameter: 900 *μ*m × 6 *μ*m), an axonal cable (1,060 *μ*m × 1 *μ*m), and a cylindrical somatic compartment (40 *μ*m × 20 *μ*m). The axonal cable included a proximal AIS (30 *μ*m), a distal AIS (30 *μ*m), and the axon (1,000 *μ*m).

The passive model properties were uniform along the model neuron: specific membrane capacitance 1 *μ*F/cm^2^, specific membrane resistance 10 kΩ cm^2^, and axial resistivity 150 Ω cm. The resting membrane potential equaled the leak reversal potential, which was set to -70 mV. The active model properties included transient sodium and delayed rectifier potassium conductances. Channel models were taken from [37, ModelDB accession number 2796], with parameter values corresponding to hippocampal pyramidal neurons. Active currents were present in all compartments (densities given in parentheses): Na-channel conductance in the soma and the dendrite (0.02 S/cm^2^), in the proximal AIS and the axon (0.04 S/cm^2^), and in the distal AIS (0.1 S/cm^2^); K-channel conductance in the soma and the dendrite (0.05 S/cm^2^), in the proximal and distal AIS (0.25 S/cm^2^), and in the axon (0.125 S/cm^2^). Additionally, the activation and inactivation curves of the Na-channels in the distal AIS and in the axon were shifted by 10 mV in hyperpolarizing direction compared to the activation and inactivation curves of Na-channels in the dendrite, the soma, and the proximal AIS.

To elicit spiking activity in the model, rectangular current stimuli of 50 ms duration were applied at the soma. The resulting somatic event amplitude was measured from the voltage at the maximum of its second derivative (i.e., maximum curvature) to the peak voltage. However, if there was no AP occurring at the AIS (detected as not crossing a voltage threshold of -20 mV), the somatic amplitude was not plotted (white regions in the heat maps). The input capacitance (Fig 3E) was calculated in the same way as in the passive-membrane model (see above).

Voltage traces shown in S2 Fig were generated in a model with default parameters, except the length of the proximal AIS, which was set to 100 *μ*m instead of the default 30 *μ*m, so that all event types (spikelet, sh-AP, fb-AP) could be produced. In S2C Fig, the dynamics of sodium channel inactivation was “frozen” to the steady-state value at -70 mV by setting the time constant of inactivation to a very large value (10^5^ ms).

Numerical simulations were performed using the NEURON simulation environment [55], with the NEURON module embedded in Python [60].

## Supporting Information

S1 Fig Fast sodium channel inactivation does not control spikelet generation in the detailed model from Fig 1. Spiking thresholds are commonly modulated by (fast) sodium channel inactivation [20]. In this context, spikelet generation could be theoretically supported by several mechanisms that restrict the soma from reaching the firing threshold, including: (1) larger somatic sodium channel inactivation, increasing the somatic firing threshold; (2) weaker Na_V_1.6 channel inactivation at the AIS during spikelets, resulting in lower AP threshold at the AIS and, thus, larger threshold difference between the AIS and the soma; and (3) larger inactivation of proximal axonal Na_V_1.2 channels, leading to smaller axial currents and, therefore, less somatic depolarization. This figure demonstrates that none of these mechanisms does account for spikelet generation in our model: AP thresholds at the soma (B1) and at the AIS (B2) are virtually identical for APs and spikelets, and so are the initial phases of the axial currents, corresponding to the currents from the AP initiated at the AIS (B3). Also the inactivation of somatic sodium channels is similar for spikelets and APs (B5). Note that the larger axial current during the late AP phase (compared to the smaller axial current during the spikelet, see B3) reflects the larger recruitment of proximal AIS sodium channels that results from stronger somatic depolarization due to stronger and longer-lasting input leading to APs as compared to spikelets. See also Fig 1H. A: Morphology of the model neuron and location of the inputs and recording sites as in Fig 1. B: AP- and spikelet-triggered averages (solid and dashed lines, respectively), aligned to the time of crossing the voltage threshold in the AIS (vertical dashed line), as in Fig 1. B1: Mean somatic AP (solid line) and mean somatic spikelet (dashed line) waveform. B2: Mean AP waveforms at the AIS for somatic APs (solid line) and somatic spikelets (dashed line). B3: Mean axial currents entering the soma from the axon hillock during somatic APs (solid line) and spikelets (dashed line). Note that the first phase of the axial current, around the AP onset (vertical dashed line), is identical for APs and spikelets. B4: Mean activation variable of somatic sodium channels during APs (solid line) and spikelets (dashed line). B5: Mean somatic sodium channel inactivation during APs (solid line) and spikelets (dashed line). B6: The participation of somatic sodium channels is substantial during APs (solid line), but much smaller during spikelets (dashed line).
(TIF)

S2 Fig Fast sodium channel inactivation does not determine the somatic threshold of APs and spikelets in the simple model from Fig 3. Unlike in the detailed model shown in Fig 1, the threshold of spikelets appears smaller than the threshold of the sh-APs in the model with reduced morphology (Fig 3B). This might suggest that an additional mechanism, besides the input amplitude, might control the generation of spikelets versus APs for a given parameter set. However, the phase plots in B4 demonstrate that the threshold at the AIS was virtually identical for all three event types (compare curves within square box), but the maximum slope and peak voltage are larger for fb-APs than sh-APs, suggesting that more sodium current is generated during fb-APs than during sh-APs. Simulations with frozen dynamics of sodium channel inactivation, shown in C, indeed abolished the differences in the somatic waveforms (i.e., maximum amplitudes and slopes), but the somatic threshold differences remained. This result implies that sodium channel inactivation is not responsible for the observed voltage threshold difference between the three event types. Instead, the lower threshold of spikelets in these simulations is caused by the ongoing somatic input: During the time between the AP initiation at the AIS and AP or spikelet occurrence at the soma, the soma is further depolarized by the ongoing current injection. Because the input is larger for APs than for spikelets, the soma depolarizes more for APs than for spikelets until the axial currents from AIS arrive, so that the somatic threshold appears higher for APs than spikelets. A: Sketch of the model neuron, as in Fig 3. B: Full voltage traces used to extract the example events (boxes) in Fig 3B. Shown are traces generated with a 50-ms long somatic stimulus, recorded at the soma (solid lines) and at the distal AIS (dashed lines). The events were generated in a model with the default parameters, only the length of the proximal AIS was increased from 30 *μ*m to 100 *μ*m so that all three event types could be generated in the same model just by varying the input strength: 0.5 nA (B1, spikelet, yellow), 0.8 nA (B2, sh-AP, dark red) and 1.3 nA (B3, fb-AP, orange). B4: Phase plots for the somatic traces (solid lines) and traces at the distal AIS (dashed lines) shown in B1–B3. The threshold at the AIS is similar for all events (curves in square box). C: The same model and inputs as in B, but the dynamics of the sodium channel inactivation variable *h* was frozen to the steady-state value at -70 mV (see Methods). Shown are voltage traces (C1) recorded at the soma (solid lines) and at the AIS (dashed lines) and the corresponding phase plots (C2). Note that somatic spikelets do not occur here because the AP at the AIS does not repolarize, so the soma remains depolarized beyond the threshold.
(TIF)

S3 Fig Comparison of spikelet properties generated in a single cell and in pairs of model cells coupled by gap junctions. We simulated spikelets resulting from somatic and dendritic coupling by gap junctions and compared them to the spikelets simulated in Fig 1 as well as to the spikelets recorded experimentally ([2], Fig 1G and [5]). The results demonstrate that properties of spikelets generated with somatic or very proximal dendritic gap junctions can fit spikelet properties observed experimentally. However, to reach such large amplitudes in the gap-junctional scenario, we had to hyperpolarize the somatic membrane voltage to -80 mV to prevent the postsynaptic cell from spiking. Moreover, the fast dynamics of experimentally recorded spikelets restricts the position of the putative somato-dendritic gap junctions to very proximal locations and predicts a very strong gap junctional conductance (37–45 nS), about 20 times stronger than the largest estimates for gap junctional conductances in cortical interneurons (0.2–2.1 nS, [57]). Spikelets simulated in a single cell (Fig 1) fit well to the properties of experimentally observed spikelets. A: Model schematic: single-cell spikelets from Fig 1 are initiated as APs at the AIS that fail to activate the soma. In cells coupled by a gap junction (colored symbols), an AP evoked in the presynaptic cell appears as a spikelet in the postsynaptic cell. The gap junction was located at the soma or at various positions along the main apical dendrite, up to 109 *μ*m away from soma (see Methods). B: Spikelet amplitude plotted against the maximum slope for spikelets generated in a single-cell model (black; Fig 1F), spikelets recorded in CA1 pyramidal cells *in vivo* (red; Fig 1G, [2]; gray [5]), and spikelets generated in the gap-junction coupling scenario for various strengths and positions of the gap junctions (see Methods; color code denotes the position of the gap junction, according to the schematic in A).
(TIF)
