# Supplementary figures and images for: Spikelets in Pyramidal Neurons: Action Potentials Initiated in the Axon Initial Segment That Do Not Activate the Soma
Source: PLoS Comput Biol. 2017 Jan 9;13(1):e1005237. doi: 10.1371/journal.pcbi.1005237 (PMC5221759; doi:10.1371/journal.pcbi.1005237)

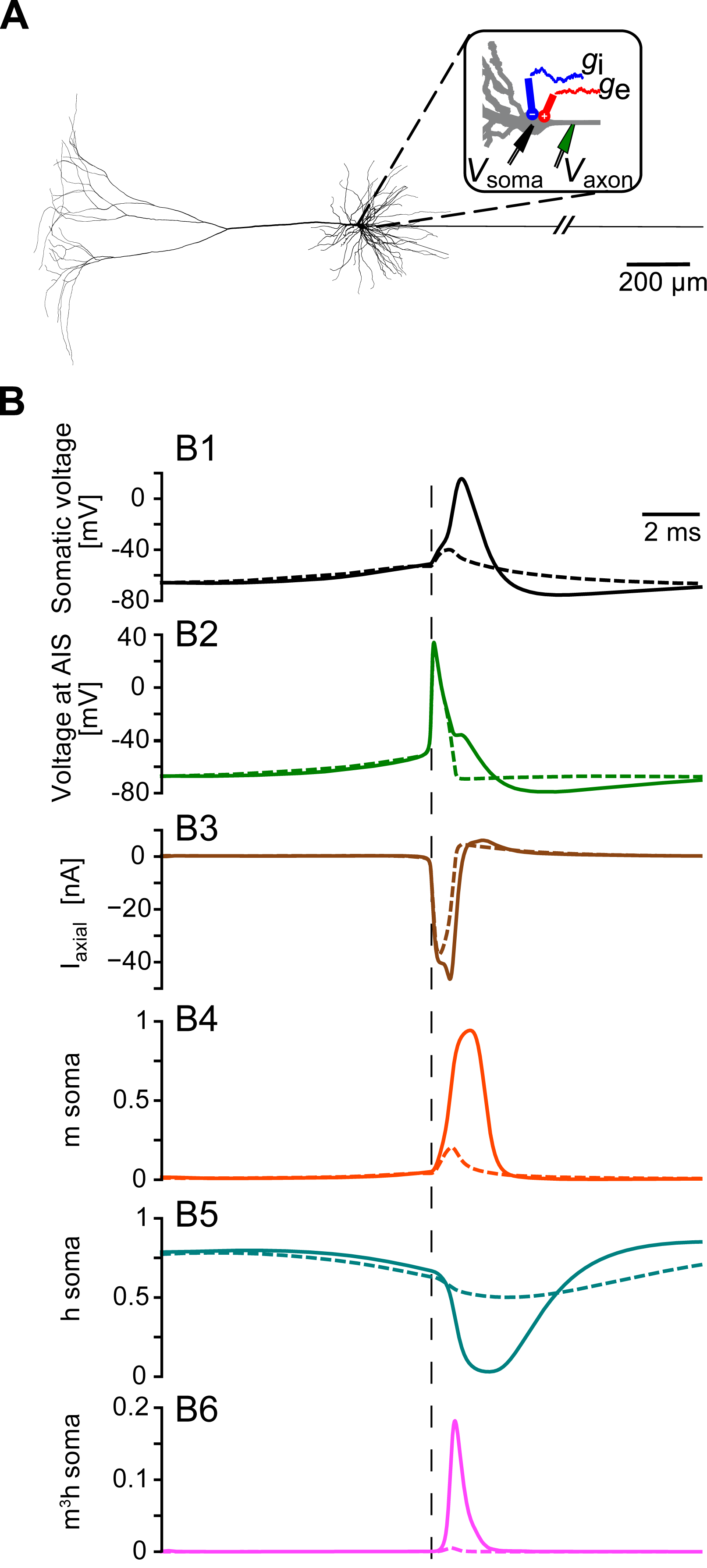

Supplement: S1 Fig — Spiking thresholds are commonly modulated by (fast) sodium channel inactivation [20]. In this context, spikelet generation could be theoretically supported by several mechanisms that restrict the soma from reaching the firing threshold, including: (1) larger somatic sodium channel inactivation, increasing the somatic firing threshold; (2) weaker NaV1.6 channel inactivation at the AIS during spikelets, resulting in lower AP threshold at the AIS and, thus, larger threshold difference between the AIS and the soma; and (3) larger inactivation of proximal axonal NaV1.2 channels, leading to smaller axial currents and, therefore, less somatic depolarization. This figure demonstrates that none of these mechanisms does account for spikelet generation in our model: AP thresholds at the soma (B1) and at the AIS (B2) are virtually identical for APs and spikelets, and so are the initial phases of the axial currents, corresponding to the currents from the AP initiated at the AIS (B3). Also the inactivation of somatic sodium channels is similar for spikelets and APs (B5). Note that the larger axial current during the late AP phase (compared to the smaller axial current during the spikelet, see B3) reflects the larger recruitment of proximal AIS sodium channels that results from stronger somatic depolarization due to stronger and longer-lasting input leading to APs as compared to spikelets. See also Fig 1H. A: Morphology of the model neuron and location of the inputs and recording sites as in Fig 1. B: AP- and spikelet-triggered averages (solid and dashed lines, respectively), aligned to the time of crossing the voltage threshold in the AIS (vertical dashed line), as in Fig 1. B1: Mean somatic AP (solid line) and mean somatic spikelet (dashed line) waveform. B2: Mean AP waveforms at the AIS for somatic APs (solid line) and somatic spikelets (dashed line). B3: Mean axial currents entering the soma from the axon hillock during somatic APs (solid line) and spikelets (dashed line). Not [file pcbi.1005237.s001.tif]

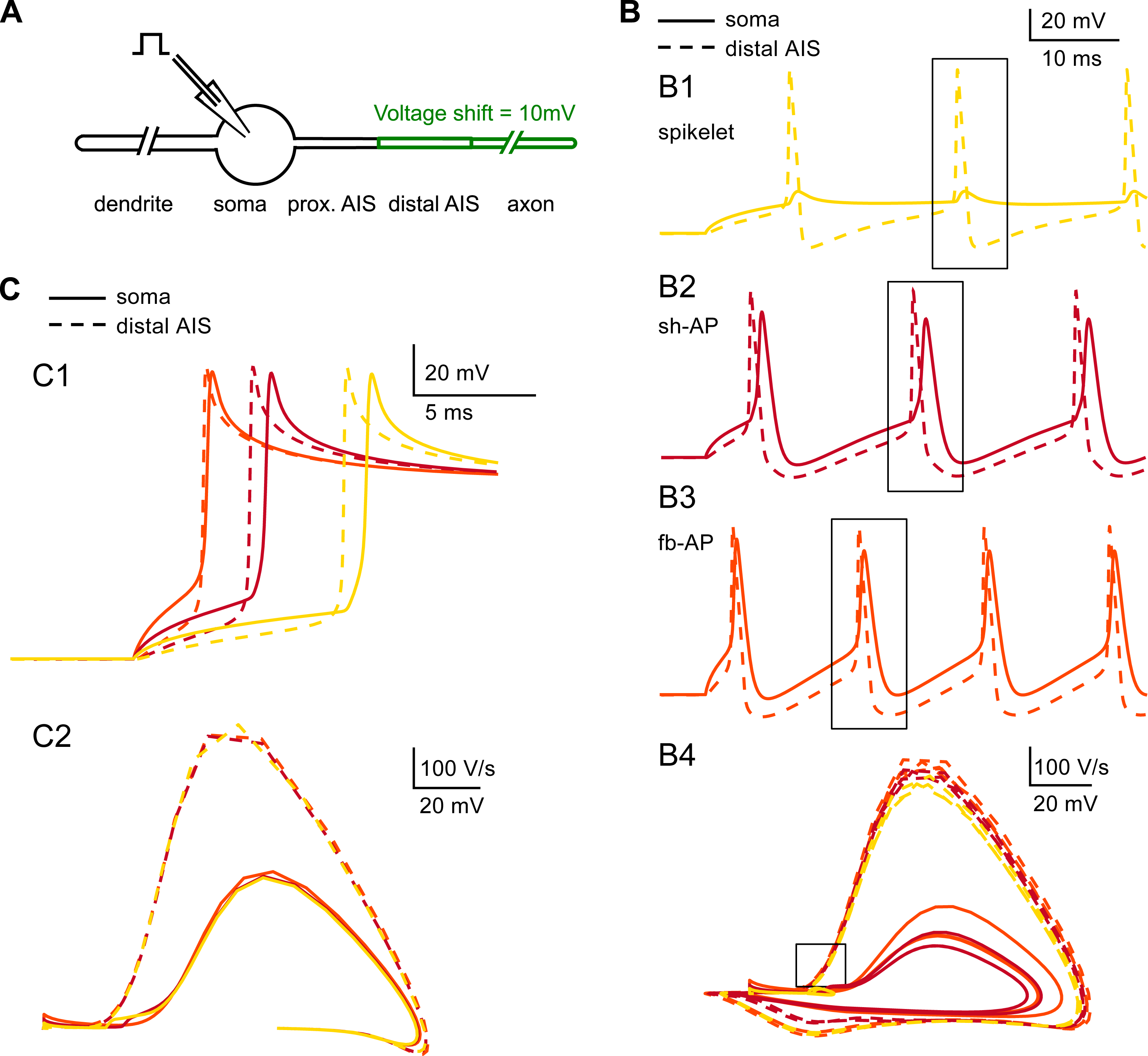

Supplement: S2 Fig — Unlike in the detailed model shown in Fig 1, the threshold of spikelets appears smaller than the threshold of the sh-APs in the model with reduced morphology (Fig 3B). This might suggest that an additional mechanism, besides the input amplitude, might control the generation of spikelets versus APs for a given parameter set. However, the phase plots in B4 demonstrate that the threshold at the AIS was virtually identical for all three event types (compare curves within square box), but the maximum slope and peak voltage are larger for fb-APs than sh-APs, suggesting that more sodium current is generated during fb-APs than during sh-APs. Simulations with frozen dynamics of sodium channel inactivation, shown in C, indeed abolished the differences in the somatic waveforms (i.e., maximum amplitudes and slopes), but the somatic threshold differences remained. This result implies that sodium channel inactivation is not responsible for the observed voltage threshold difference between the three event types. Instead, the lower threshold of spikelets in these simulations is caused by the ongoing somatic input: During the time between the AP initiation at the AIS and AP or spikelet occurrence at the soma, the soma is further depolarized by the ongoing current injection. Because the input is larger for APs than for spikelets, the soma depolarizes more for APs than for spikelets until the axial currents from AIS arrive, so that the somatic threshold appears higher for APs than spikelets. A: Sketch of the model neuron, as in Fig 3. B: Full voltage traces used to extract the example events (boxes) in Fig 3B. Shown are traces generated with a 50-ms long somatic stimulus, recorded at the soma (solid lines) and at the distal AIS (dashed lines). The events were generated in a model with the default parameters, only the length of the proximal AIS was increased from 30 μm to 100 μm so that all three event types could be generated in the same model just by varying the input strength: 0.5 n [file pcbi.1005237.s002.tif]

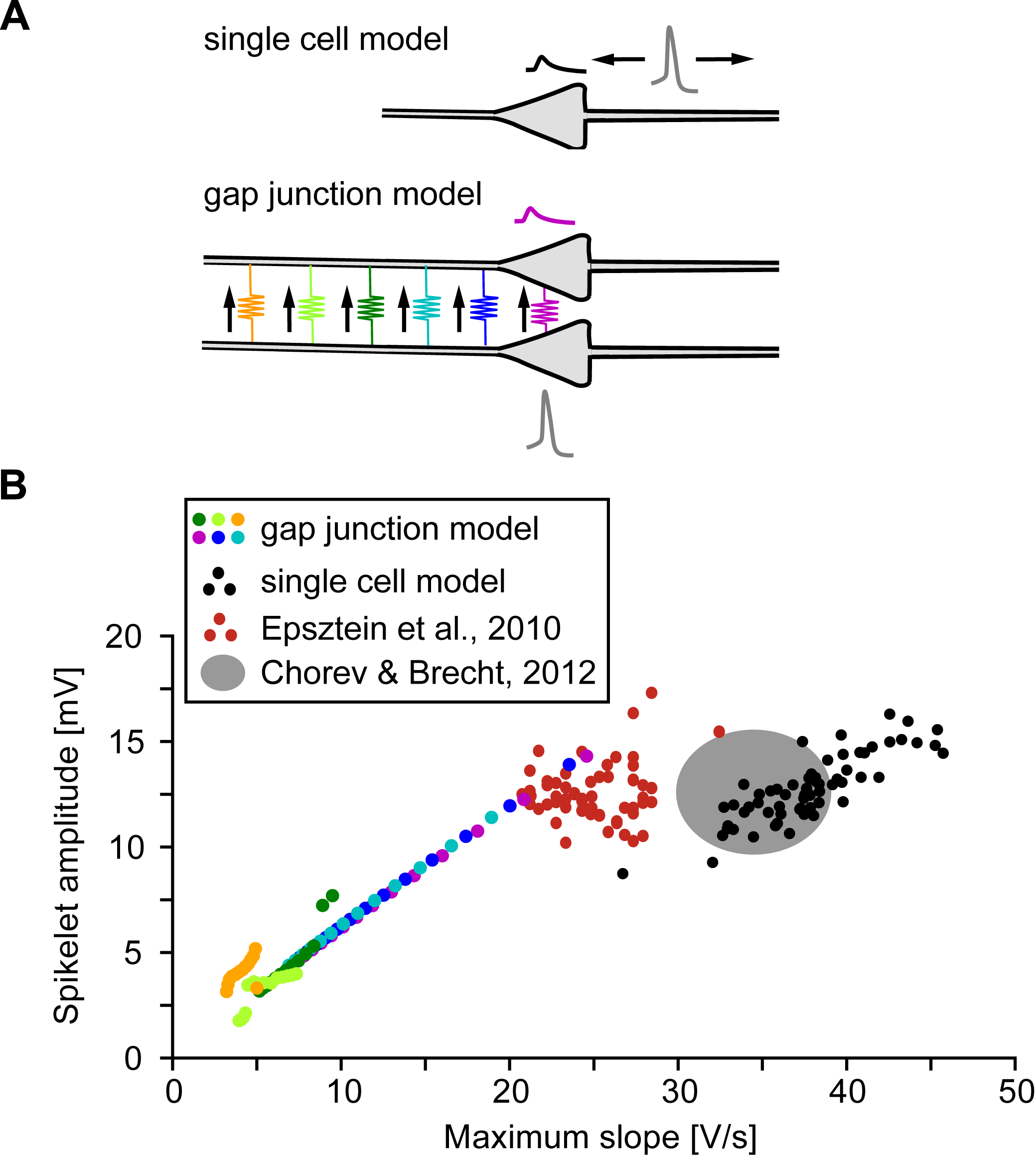

Supplement: S3 Fig — We simulated spikelets resulting from somatic and dendritic coupling by gap junctions and compared them to the spikelets simulated in Fig 1 as well as to the spikelets recorded experimentally ([2], Fig 1G and [5]). The results demonstrate that properties of spikelets generated with somatic or very proximal dendritic gap junctions can fit spikelet properties observed experimentally. However, to reach such large amplitudes in the gap-junctional scenario, we had to hyperpolarize the somatic membrane voltage to -80 mV to prevent the postsynaptic cell from spiking. Moreover, the fast dynamics of experimentally recorded spikelets restricts the position of the putative somato-dendritic gap junctions to very proximal locations and predicts a very strong gap junctional conductance (37–45 nS), about 20 times stronger than the largest estimates for gap junctional conductances in cortical interneurons (0.2–2.1 nS, [57]). Spikelets simulated in a single cell (Fig 1) fit well to the properties of experimentally observed spikelets. A: Model schematic: single-cell spikelets from Fig 1 are initiated as APs at the AIS that fail to activate the soma. In cells coupled by a gap junction (colored symbols), an AP evoked in the presynaptic cell appears as a spikelet in the postsynaptic cell. The gap junction was located at the soma or at various positions along the main apical dendrite, up to 109 μm away from soma (see Methods). B: Spikelet amplitude plotted against the maximum slope for spikelets generated in a single-cell model (black; Fig 1F), spikelets recorded in CA1 pyramidal cells in vivo (red; Fig 1G, [2]; gray [5]), and spikelets generated in the gap-junction coupling scenario for various strengths and positions of the gap junctions (see Methods; color code denotes the position of the gap junction, according to the schematic in A). (TIF) [file pcbi.1005237.s003.tif]
